# Lower platelet mitochondrial function in severe septic patients than in controls

**DOI:** 10.1186/cc14106

**Published:** 2015-03-16

**Authors:** L Lorente, M Martin, J Blanquer, J Solé-Violán, L Labarta, C Díaz, A Jiménez, E López-Gallardo, J Montoya, E Ruiz-Pesini

**Affiliations:** 1 Hospital Universitario de Canarias, La Laguna, Tenerife, Spain; 2 Hospital Universitario Nuestra Señora Candelaria, Santa Cruz, Tenerife, Spain; 3 Hospital Clínico Universitario de Valencia, Spain; 4 Hospital Universitario Dr Negrín, Las Palmas de Gran Canaria, Spain; 5 Hospital San Jorge, Huesca, Spain; 6 Hospital Insular, Las Palmas de Gran Canaria, Spain; 7 Universidad de Zaragoza, Spain

## Introduction

The oxidative phosphorylation system (OXPHOS) in septic patients has been scarcely analyzed in studies of small sample size and the results are apparently inconsistent. Previously, including 96 severe septic patients, we found that nonsurviving severe septic patients showed lower platelet respiratory complex IV (CIV) activity than surviving patients at the moment of severe sepsis diagnosis and during the first week of sepsis diagnosis. However, we did not examine this enzyme activity in normal individuals. Thus, the objective of this study was to compare the CIV activity between severe septic patients and healthy control individuals in a larger series of patients (including 198 severe septic patients).

## Methods

This was a prospective, multicenter, observational study in six Spanish ICUs. We obtained blood samples from 198 severe septic patients at days 1, 4 and 8 of the severe sepsis diagnosis and from 96 sex-matched and age-matched healthy control individuals and determined platelet CIV activity/protein quantity. The endpoint of the study was 30-day mortality.

## Results

We found that severe septic patients showed lower CIV activity/protein quantity than controls at day 1 (*P *< 0.001), day 4 (*P *< 0.001) and day 8 (*P *< 0.001) of severe sepsis diagnosis. Survivor severe septic patients (*n *= 130) showed lower CIV activity/protein quantity than controls at day 1 (*P *< 0.001), day 4 (*P *< 0.001) and day 8 (*P *< 0.001) of severe sepsis diagnosis. In addition, nonsurvivor severe septic patients (*n *= 68) showed lower CIV activity/protein quantity than controls at day 1 (*P *< 0.001), day 4 (*P *< 0.001) and day 8 (*P *< 0.001) of severe sepsis diagnosis. Besides, nonsurvivor severe septic patients showed lower CIV activity/protein quantity than survivor ones at day 1 (*P *< 0.001), day 4 (*P *< 0.001) and day 8 (*P *< 0.001) of severe sepsis diagnosis.

## Conclusion

The major finding of our work, that represents the largest series of severe septic patients with data on OXPHOS function, was that survivor and nonsurvivor severe septic patients showed lower platelet CIV activity than healthy controls during the first week of severe sepsis diagnosis.

